# Elevated Blood Lead Levels Associated with Retained Bullet Fragments — United States, 2003–2012

**DOI:** 10.15585/mmwr.mm6605a2

**Published:** 2017-02-10

**Authors:** Debora Weiss, Carrie D. Tomasallo, Jon G. Meiman, Walter Alarcon, Nathan M. Graber, Kristine M. Bisgard, Henry A. Anderson

**Affiliations:** ^1^Epidemic Intelligence Service, Division of Scientific Education and Professional Development, CDC; ^2^Bureau of Environmental and Occupational Health, Wisconsin Department of Health Services; ^3^National Institute of Occupational Safety and Health, CDC; ^4^New York State Department of Health; ^5^Center for Surveillance, Epidemiology and Laboratory Services, CDC.

An estimated 115,000 firearm injuries occur annually in the United States, and approximately 70% are nonfatal (*1*). Retained bullet fragments (RBFs) are an infrequently reported, but important, cause of lead toxicity; symptoms are often nonspecific and can appear years after suffering a gunshot wound (*2*,*3*). Adult blood lead level (BLL) screening is most commonly indicated for monitoring of occupational lead exposure; routine testing of adults with RBFs is infrequent (*3*). States collaborate with CDC’s National Institute for Occupational Safety and Health (NIOSH) to monitor elevated BLLs through the Adult Blood Lead Epidemiology and Surveillance (ABLES) program (*4*,*5*). To help assess the public health burden of RBFs, data for persons with BLLs ≥10 *μ*g/dL reported to ABLES during 2003–2012 were analyzed. An RBF-associated case was defined as a BLL ≥10 *μ*g/dL in a person with an RBF. A non-RBF–associated case was defined as a BLL ≥10 *μ*g/dL without an RBF. During 2003–2012, a total of 145,811 persons aged ≥16 years with BLLs ≥10 *μ*g/dL were reported to ABLES in 41 states. Among these, 457 RBF-associated cases were identified with a maximum RBF-associated BLL of 306 *μ*g/dL. RBF-associated cases accounted for 0.3% of all BLLs ≥10 *μ*g/dL and 4.9% of BLLs ≥80 *μ*g/dL. Elevated BLLs associated with RBFs occurred primarily among young adult males in nonoccupational settings. Low levels of suspicion of lead toxicity from RBFs by medical providers might cause a delay in diagnosis (*3*). Health care providers should inquire about an RBF as the potential cause for lead toxicity in an adult with an elevated BLL whose lead exposure is undetermined.

At BLLs ≥10 *μ*g/dL, hypertension, kidney dysfunction, possible subclinical neurocognitive deficits, and adverse reproductive outcomes (including spontaneous abortion and reduced birthweight) can occur (*6*,*7*). Decreased renal function has been documented in association with BLLs <5 *μ*g/dL, and an increased risk for hypertension and essential tremor at BLLs <10 *μ*g/dL (*8*).

States collaborate with NIOSH to conduct blood lead surveillance through the ABLES program (*4*). In 2009, for the purposes of surveillance and risk factor ascertainment, the Council of State and Territorial Epidemiologists (CSTE), NIOSH, and ABLES lowered the cutoff for an elevated BLL from ≥25 *μ*g/dL to ≥10 *μ*g/dL of lead in a venous sample of whole blood (*4*,*9*). In 2015, NIOSH and CSTE further reduced the case definition for an elevated BLL to 5 *μ*g/dL (*4*). States participating in ABLES require health care providers and laboratories to report blood lead test results to the state health department. Certain states require all BLLs to be reported, whereas other states require reporting of BLLs ≥10, ≥25, or ≥40 *μ*g/dL (*4*). States follow up to identify the industry in which the affected person is employed and determine whether the exposure source is occupational, nonoccupational, or both, and provide a short narrative describing the activity during which the lead exposure occurred. Screening for adult lead exposure focuses on settings where occupational lead exposure is likely; adults with RBFs are not routinely tested for lead (*3*).

CDC analyzed data for adults with BLLs ≥10 *μ*g/dL reported by the ABLES program during 2003–2012. An RBF-associated case of elevated BLL was defined as a BLL ≥10 *μ*g/dL in a person aged ≥16 years with ≥1 RBFs at the time of blood collection. A non-RBF–associated case was defined as a BLL ≥10 *μ*g/dL in a person without an RBF or bullet fragment in a person aged ≥16 years. RBF cases were identified as persons coded with “retained bullets (gunshot wounds)” in the ABLES database. If a person had multiple blood lead tests during the study period (2003–2012), only the highest BLL was included. In 2003, a total of 36 states reported BLLs ≥25 *μ*g/dL, and 20 of these states also reported BLLs 10–24 *μ*g/dL. In 2012, 41 states reported BLLs ≥25 *μ*g/dL, and 38 of these states also reported BLLs 10–24 *μ*g/dL.

During 2003–2012, a total of 41 state ABLES programs reported 145,811 adults with elevated BLLs from all causes, including 349 (0.2%) with BLLs ≥80 *μ*g/dL. RBF-associated cases accounted for 457 (0.3%) of adults with elevated BLLs, but 17 (4.9%) of adults with BLLs ≥80 *μ*g/dL (Figure); the maximum recorded RBF-associated BLL was 306 *μ*g/dL. Furthermore, RBF-associated cases were overrepresented among persons with BLLs ≥80 *μ*g/dL, compared with non-RBF–associated cases: 17 (3.7%) of 457 patients with RBF-associated elevated BLLs had BLLs ≥80 *μ*g/dL, compared with 332 (0.2%) of 145,354 patients with non-RBF–associated elevated BLLs.

**FIGURE:**
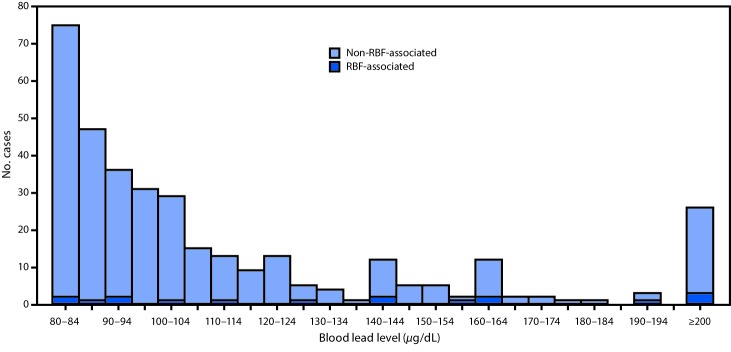
Number of patients with and without a retained bullet fragment (RBF) among persons with blood lead levels ≥80 *μ*g/dL — United States, 2003–2012

Among 457 RBF-associated cases, 195 (42.7%) occurred among persons aged 16–24 years (Table), whereas only 11.8% (n = 17,151) of 145,354 non-RBF–associated cases occurred in this age group (Table). In contrast, 36,462 (25.1%) of the non-RBF–associated cases occurred in persons aged 35–44 years (Table), compared with 72 (15.8%) of 457 RBF-associated cases (Table). Males accounted for 83.5% of RBF-associated cases and 89.9% of non-RBF–associated cases. Sex was not listed for two (0.4%) persons among RBF-associated cases and 1,837 (1.3%) among non-RBF–associated cases.

**TABLE:** Number of retained bullet fragment–associated cases, by age and highest reported blood lead level — United States, 2003–2012

Age group (yrs)	Blood lead level (*µ*g/dL)							
	10–24	25–39	40–59	60–79	80–199	200–299	≥300	Total
	No. (%)	No. (%)	No. (%)	No. (%)	No. (%)	No. (%)	No. (%)	No. (%)
**16–24**	132 (48.9)	48 (41.4)	13 (30.9)	2 (16.7)	—	—	—	**195 (42.7)**
**25–34**	39 (14.4)	20 (17.2)	8 (19.0)	1 (8.3)	1 (7.1)	—	—	**69 (15.1)**
**35–44**	34 (12.6)	17 (14.7)	7 (16.7)	7 (58.3)	5 (35.7)	1 (50.0)	1 (100)	**72 (15.8)**
**45–54**	28 (10.4)	16 (13.8)	6 (14.3)	2 (16.7)	3 (21.4)	1 (50.0)	—	**56 (12.3)**
**55–64**	29 (10.7)	10 (8.6)	5 (11.9)	—	5 (35.7)	—	—	**49 (10.7)**
**≥65**	8 (3.0)	5 (4.4)	3 (7.1)	—	—	—	—	**16 (3.5)**
**Total**	**270 (59.1)**	**116 (25.4)**	**42 (9.2)**	**12 (2.6)**	**14 (3.1)**	**2 (<1)**	**1 (<1)**	**457 (100.0)**

The majority of persons with RBF-associated elevated BLLs did not report an occupational exposure. Among the 457 RBF-associated cases for which exposure source was known, 446 (97.6%) were nonoccupational, two (0.4%) persons were employed in police protection or amusement and recreation industries, and nine (2.0%) had both occupational and nonoccupational exposures coded, although the occupation was not available. In contrast, among 77,770 (54%) non-RBF–associated cases with a known exposure source, 5,113 (6.6%) were nonoccupational and 261 (0.2%) had both occupational and nonoccupational exposures coded. Among 270 (59.1%) of 457 RBF-associated cases and 93,273 (64.2%) of 145,354 non-RBF–associated cases, the highest recorded BLL was 10–24 *μ*g/dL.

## Discussion

Symptoms resulting from elevated BLLs can vary widely and are often nonspecific, including fatigue, abdominal pain, and memory loss (*2*,*6*). As of 2004, fewer than 100 cases of lead toxicity caused by RBFs had been reported in the medical literature (*3*). During 2003–2012, elevated BLLs associated with RBFs constituted 0.3% of all elevated BLLs and 4.9% of BLLs ≥80 *μ*g/dL. Elevated BLLs associated with RBFs occurred predominantly among males aged 16–24 years in nonoccupational settings. The population identified in this study differs from the population exposed to lead in occupational settings, where cases are identified through a mechanism of routine lead exposure screening. However, adult males without an occupational exposure, including those with RBFs, would likely only be screened if they seek care for symptoms related to elevated BLLs, or as part of routine care for other purposes, if suspicion is raised by a medical provider. In addition, a low index of suspicion of lead toxicity by medical providers might result in a delay in diagnosis, and patients might receive multiple incorrect diagnoses before receiving correct assessment and treatment (*2*). Furthermore, BLLs can fluctuate in persons with RBFs. A person with a low BLL at the time of testing can have an increase in BLL and become symptomatic when RBFs migrate, such as into a joint space (*3*,*10*).

The findings in this report are subject to at least four limitations. First, not all states report to ABLES, and persons with RBFs are often not tested; therefore, these data should be considered minimum estimates of the magnitude of the problem. Second, the reporting requirement varies by state and ranges from requiring reporting of all BLLs to only those ≥40 *μ*g/dL. Third, before 2007, work-related RBFs were not systematically identified through ABLES; identification of adults at risk for lead exposure is limited primarily to certain groups at high risk and universal blood lead screening is not standard practice. Finally, only some states provided 10–24 *μ*g/dL BLL data. The possibility exists that some reporting states might not have investigated patients with BLLs 10–24 *μ*g/dL or determined the location where lead exposure occurred, thereby resulting in omission or misclassification of RBF cases.

Persons with elevated BLLs with an unknown exposure source can be queried about RBFs. Patients with RBFs might benefit from counseling on lead and its health effects, and the importance of baseline and periodic BLL monitoring (*6*,*7*).

**Summary**

**What is already known about this topic?**

Gunshot wounds cause an estimated 115,000 injuries in the United States per year, approximately 70% of which are nonfatal. Bullet removal is not routinely indicated for victims of gunshot injuries with retained bullet fragments (RBFs) unless they are a cause of immediate morbidity. Symptoms of lead toxicity are often nonspecific and can appear years after the initial injury. States participating in the Adult Blood Lead Epidemiology and Surveillance (ABLES) program require health care providers and laboratories to report blood lead level (BLL) test results to the state health department. The primary focus of adult screening is to detect occupational exposure; RBFs are a less recognized potential source of lead exposure.

**What is added by this report?**

During 2003–2012, ABLES programs in 41 states reported 145,811 persons with BLLs ≥10 *µ*g/dL. RBF-associated cases accounted for 457 (0.3%) of 145,811 persons with elevated BLLs. Among 349 persons with BLLs ≥80 *µ*g/dL, 17 (4.9%) were RBF-associated; the maximum recorded RBF-associated BLL was 306 *µ*g/dL. Elevated BLLs attributable to RBFs occurred primarily among males aged 16–24 years, whereas the greatest number of non-RBF–associated cases occurred among persons aged 35–44 years.

**What are the implications for public health practice?**

Persons with elevated BLLs with unknown lead exposure source should be asked about RBFs. Furthermore, baseline and intermittent BLL tests should be considered in persons with a history of RBFs.
